# Different treatment response to systemic corticosteroids according to white blood cell counts in severe COVID-19 patients

**DOI:** 10.1080/07853890.2022.2137736

**Published:** 2022-12-01

**Authors:** Kwang Yong Choi, Dong Hyun Kim, Kwang Nam Jin, Hyo Jin Lee, Tae Yun Park, Borim Ryu, Jung-Kyu Lee, Eun Young Heo, Deog Kyeom Kim, Hyun Woo Lee

**Affiliations:** a Division of Pulmonary and Critical Care Medicine, Department of Internal Medicine, Seoul National University College of Medicine, Seoul Metropolitan Government-Seoul National University Boramae Medical Center, Seoul, South Korea; b Department of Radiology, Seoul Metropolitan Government-Seoul National University Boramae Medical Center, Seoul, South Korea; c Biomedical Research Institute, Seoul Metropolitan Government-Seoul National University Boramae Medical Center, Seoul, South Korea

**Keywords:** COVID-19, SARS-CoV-2, pneumonia, viral, leukocyte count, glucocorticoids, treatment outcome

## Abstract

**Background:**

Limited data are available in COVID-19 patients on the prediction of treatment response to systemic corticosteroid therapy based on systemic inflammatory markers. There is a concern whether the response to systemic corticosteroid is different according to white blood cell (WBC) counts in COVID-19 patients. We aimed to assess whether WBC count is related with the clinical outcomes after treatment with systemic corticosteroids in severe COVID-19.

**Methods:**

We conducted a retrospective cohort study and analysed the patients hospitalised for severe COVID-19 and received systemic corticosteroids between July 2020 and June 2021. The primary endpoint was to compare the composite poor outcome of mechanical ventilation, extracorporeal membrane oxygenation, and mortality among the patients with different WBC counts.

**Results:**

Of the 585 COVID-19 patients who required oxygen supplementation and systemic corticosteroids, 145 (24.8%) belonged to the leukopoenia group, 375 (64.1%) belonged to the normal WBC group, and 65 (11.1%) belonged to the leukocytosis group. In Kaplan-Meier curve, the composite poor outcome was significantly reduced in leukopoenia group compared to leukocytosis group (log-rank *p*-value < 0.001). In the multivariable Cox regression analysis, leukopoenia group was significantly associated with a lower risk of the composite poor outcome compared to normal WBC group (adjusted hazard ratio [aHR] = 0.32, 95% CI 0.14–0.76, *p*-value = 0.009) and leukocytosis group (aHR = 0.30, 95% CI = 0.12–0.78, *p*-value = 0.013). There was no significant difference in aHR for composite poor outcome between leukocytosis and normal WBC group.

**Conclusion:**

Leukopoenia may be related with a better response to systemic corticosteroid therapy in COVID-19 patients requiring oxygen supplementation.

**KEY MESSAGES**
- In severe COVID-19 treated with systemic corticosteroids, patients with leukopoenia showed a lower hazard for composite poor outcome compared to patients with normal white blood cell counts or leukocytosis.
- Leukopoenia may be a potential biomarker for better response to systemic corticosteroid therapy in COVID-19 pneumonia.

## Introduction

Systemic corticosteroid therapy has been a critical component for the management of severe coronavirus disease 2019 (COVID-19) with respiratory failure [1]. In previous randomised controlled trials (RCTs), administration of systemic corticosteroids improved mortality and increased ventilator free days in COVID-19 patients [2,3]. Especially, dexamethasone had significant benefits in the COVID-19 patients who required oxygen supplementation or mechanical ventilation [2]. However, the mortality rate of severe COVID-19 patients was still as high as 26.6%, even though systemic corticosteroids were used [1]. Therefore, we need to differentiate the COVID-19 patients with heterogeneous responses to systemic corticosteroids.

To provide precise and individualised management for the COVID-19 patients, it is essential to identify the subgroups who either responds well or poorly to standard treatment consisting mainly of systemic corticosteroids. Considering that the efficacy of corticosteroids on COVID-19 relies on their anti-inflammatory or immunomodulatory effects [4], systemic inflammatory biomarkers may have a potential role to predict the response to corticosteroids. There have been few studies that investigated inflammatory phenotypes related with the response to systemic corticosteroids. A recent study showed that systemic corticosteroids may be less effective in the critically-ill COVID-19 patients with hypo-inflammatory phenotypes [5]. A better clinical response to systemic corticosteroids has been expected in hyper-inflammatory phenotypes than hypo-inflammatory phenotypes [6]. Among various systemic inflammatory markers, white blood cell (WBC) has been considered as an important indicator to differentiate hyper- and hypo-inflammatory phenotypes in acute respiratory distress syndrome (ARDS) [7,8]. WBC count was related with cytokine storm syndrome, higher severity, and poor prognosis in COVID-19 [9–11]. Although WBC count is an important biologic marker for systemic inflammation, it has not been figured out whether treatment response to systemic corticosteroid for severe COVID-19 is different according to WBC count.

Therefore, we investigated whether the clinical outcomes after treatment with systemic corticosteroid are different according to the WBC counts in COVID-19 patients who required oxygen therapy.

## Methods

We reported the present study following the guidance of the Strengthening the Reporting of Observational Studies in Epidemiology (STROBE) statement.

### Study design and participants

We conducted a retrospective cohort study and analysed the patients who were hospitalised for severe COVID-19 at Boramae Medical Centre between July 2020 and June 2021. All the eligible patients were: (1) age >18 years old, (2) diagnosed with COVID-19 through quantitative reverse transcription-polymerase chain reaction (RT-qPCR) assays using respiratory tract samples, (3) treated with systemic corticosteroids for severe COVID-19. Systemic corticosteroids were administrated when the COVID-19 patients required oxygen therapy or had a significant desaturation (room-air percutaneous arterial oxygen saturation [SpO_2_] < 90%). Exclusion criteria were immunosuppressive conditions including haematologic malignancy or current chemotherapy for active cancer, administration of systemic corticosteroids or immunosuppressive drugs for other conditions, pregnant status, and terminal stage disease with documented Do Not Resuscitate order.

### Variables

Baseline demographic data were collected including age, sex, body mass index (BMI), cigarette smoking status, cigarette pack-years, and comorbidities (hypertension, diabetes, chronic obstructive pulmonary disease (COPD), asthma, interstitial lung disease (ILD), history of tuberculosis, lung cancer, other malignancy, chronic kidney disease, chronic liver disease, cardiovascular disease, cerebrovascular disease, cognitive disorder, and connective tissue disease). Symptomatic manifestation data was systematically recorded in electronic medical records including smell or taste abnormalities, myalgia, sore throat, cough, sputum, chest discomfort, dyspnoea, fever, rhinorrhea or nasal congestion, and diarrhoea. Information on vital signs and parameters for oxygen status including systolic blood pressure (SBP), diastolic blood pressure (DBP), heart rate (HR), respiratory rate (RR), SpO_2_, oxygen requirement (L/min), fraction of inspired oxygen (FiO_2_), SpO_2_/FiO_2_ (SF ratio), and ROX index (SF ratio/RR) were obtained. Laboratory and radiologic examinations were routinely performed for the hospitalised patients with oxygen requirement including complete blood count (CBC) with differential count, haemoglobin, platelet, C-reactive protein (CRP), procalcitonin, lactate dehydrogenase (LDH), D-dimer, troponin I, NT-proB-type natriuretic peptide (NT-proBNP), blood urea nitrogen (BUN), creatinine, aspartate transaminase (AST), alanine transaminase (ALT), total bilirubin, prothrombin time international normalised ratio (PT INR), and chest X-ray. Information on treatment including Lopinavir/Ritonavir, hydroxychloroquine, remdesivir, antibiotics, duration or cumulative dose of corticosteroids were collected. Information on clinical outcomes including high flow nasal cannula (HFNC), mechanical ventilation (MV), extracorporeal membrane oxygenation (ECMO), mortality, and hospital length of stay (LOS) were obtained.

### Study outcomes

The primary endpoint was to compare the composite poor outcome including mechanical ventilation, ECMO and mortality among three groups classified based on different WBC counts: leukopoenia, normal leukocyte, and leukocytosis groups. The patients with WBC <4000 were defined as leukopoenia group, those with WBC from 4000 to 9999 were defined as normal WBC group, and those with WBC ≥10,000 were defined as leukocytosis group.

### Statistical analyses

Demographic information, symptomatic manifestation, vital signs and parameters for oxygen status, laboratory and radiologic results, treatments and clinical outcomes were compared among the different three groups. We analysed categorical variables using Pearson’s chi-squared test or the Fisher’s exact test and continuous variables using ANOVA test or Kruskal-Wallis test. To analyse linear trend among the three groups, the Cochran-Armitage test was used for categorical variables, and linear regression analysis was used for continuous variables. We conducted repeated-measure ANOVA tests to evaluate the changes of WBC counts. We performed survival analysis with Kaplan-Meier estimation. To minimise the potential risk of bias due to confounding factors, multivariate Cox regression analysis was conducted with statistically significant covariables selected through backward elimination. Statistical significance was determined as *p*-values <0.05. All the statistical analyses were conducted using R statistical software, version 4.1.1 (R Core Team, [2021], Vienna, Austria).

### Ethics

This study was conducted in accordance with the principles of the Declaration of Helsinki. The Institutional Review Board of Seoul Metropolitan Government-Seoul National University Boramae Medical Centre approved the study protocol waived the need for informed consent for access to the electronic medical records (IRB No. 30-2022-40).

## Results

### Baseline characteristics

**Figure 1.**
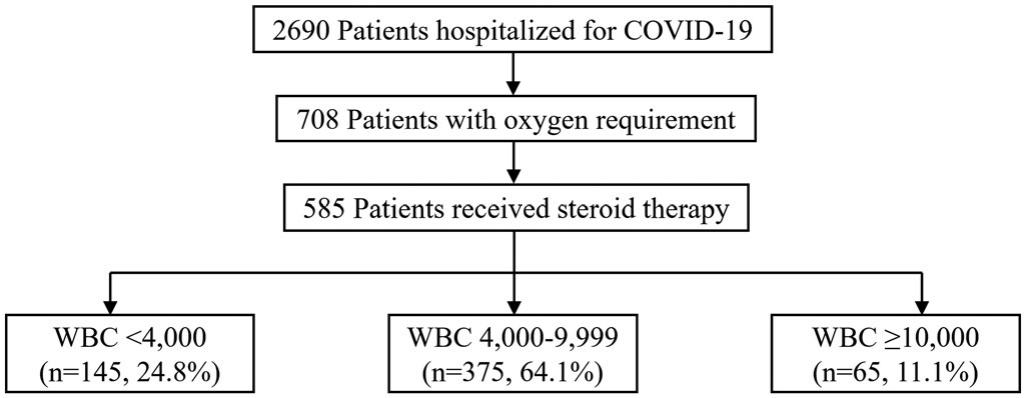
Flow diagram of the study population.

Of the 2690 patients hospitalised for COVID-19 infection, 708 patients required oxygen therapy. Among these patients with oxygen requirement, 585 patients who received systemic corticosteroid therapy for severe COVID-19 were eligible. In the eligible study patients, 145 (24.8%) belonged to the leukopoenia group, 375 (64.1%) belonged to the normal WBC group, and 65 (11.1%) belonged to the leukocytosis group (Figure 1). The time intervals from symptom onset, diagnosis of COVID-19, and hospital admission to oxygen requirement were median 7 (interquartile range [IQR] = 5–10), 3 (IQR = 1–6), and 1 (IQR = 1–3) days, respectively.

The baseline characteristics of the included patients are presented in Table 1. Leukocytosis group was older with mean age of 74.2 years compared to 68.1 and 68.5 years in leukopoenia and normal WBC group, respectively. The proportion of hypertension and asthma showed a positive linear trend according to the WBC status. Otherwise, there were no significant differences across the groups.

**Table 1.** Baseline demographic characteristics according to different white blood cell counts.

	WB*C* < 4000 (*n* = 145)	WBC 4000–9999 (*n* = 375)	WB*C* ≥ 10,000 (*n* = 65)	*p*-Value	*p*-Trend*
Age, years	68.1 (±14.3)	68.5 (±14.2)	74.2 (±12.0)	0.006	0.019
Ag*e* > 65, *n* (%)	181 (48.3)	62 (42.8)	43 (66.2)	0.007	0.103
Female, *n* (%)	74 (51.0)	160 (42.7)	28 (43.1)	0.218	0.148
Body mass index, kg/m^2^	24.2 (±3.3)	25.0 (±4.3)	23.2 (±4.3)	0.002	0.603
Ever smoker, *n* (%)	10 (6.9)	32 (8.5)	6 (9.2)	0.424	0.787
Smoking intensity, pack-years	2.2 (±9.7)	1.9 (±8.7)	1.8 (±6.2)	0.942	0.743
Comorbidity, *n* (%)					
Hypertension	61 (42.1)	202 (53.9)	36 (55.4)	0.042	0.024
Diabetes mellitus	40 (27.6)	116 (30.9)	23 (35.4)	0.512	0.251
COPD	3 (2.1)	12 (3.2)	2 (3.1)	0.786	0.577
Asthma	1 (0.7)	15 (4.0)	4 (6.2)	0.077	0.025
ILD	0 (0.0)	1 (0.3)	0 (0.0)	0.755	0.815
Tuberculosis	3 (2.1)	16 (4.3)	2 (3.1)	0.469	0.477
Lung cancer	3 (2.1)	4 (1.1)	0 (0.0)	0.412	0.184
Other malignancy	17 (11.7)	26 (6.9)	9 (13.8)	0.075	0.825
Chronic kidney disease	11 (7.6)	19 (5.1)	2 (3.1)	0.351	0.150
Chronic liver disease	6 (4.1)	13 (3.5)	0 (0.0)	0.272	0.174
Cardiovascular disease	19 (13.1)	42 (11.2)	12 (18.5)	0.254	0.523
Cerebrovascular disease	25 (17.2)	44 (11.7)	7 (10.8)	0.210	0.109
Cognitive disorder	0 (0.0)	6 (1.6)	0 (0.0)	0.183	0.565
Connective tissue disease	7 (4.8)	5 (1.3)	3 (4.6)	0.042	0.383

Data are presented as *n* (%) or mean ± standard deviation.

*Categorical variables were analysed using Cochran-Armitage test, continuous variables were analysed using linear regression.

### Clinical characteristics and initial laboratory indices

The clinical characteristics and initial laboratory indices are presented in Table 2. For initial vital signs and parameters, SBP, HR, and oxygen requirement or FiO_2_ showed positive linear trends according to the WBC status while SpO_2_, SF ratio, and ROX index showed negative linear trends. For initial laboratory indices, neutrophil-to-lymphocyte ratio (NLR) showed a positive linear trend according to the WBC status while monocytes showed a negative linear trend. Platelet counts, CRP, procalcitonin, LDH, D-dimer, BUN, AST, ALT, total bilirubin, and PT INR also showed positive linear trends. In the analysis of WBC changes during 5 days of steroids, WBC count and neutrophil count increased in leukopoenia and normal WBC group, while decreased in leukocytosis group (supplemental Figure S1). In addition, lymphocyte count of leukopoenia group was more increased compared with that of leukocytosis group, although lymphocyte counts at D1 were similar. There was no difference in the presence of pneumonic infiltration in chest X-rays across the groups.

**Table 2.** Clinical manifestation according to different white blood cell counts.

	WB*C* < 4000 (*n* = 145)	WBC 4000–9999 (*n* = 375)	WB*C* ≥ 10,000 (*n* = 65)	*p*-Value	*p*-Trend*
Symptomatic manifestation, *n* (%)					
Smell or taste abnormality	26 (17.9)	73 (19.5)	7 (10.8)	0.243	0.408
Myalgia	47 (32.4)	112 (29.9)	17 (26.2)	0.651	0.360
Sore throat	36 (24.8)	73 (19.5)	8 (12.3)	0.101	0.034
Cough	95 (65.5)	238 (63.5)	38 (58.5)	0.618	0.357
Sputum	53 (36.6)	180 (48.0)	33 (50.8)	0.042	0.020
Chest discomfort	19 (13.1)	44 (11.7)	12 (18.5)	0.323	0.491
Dyspnoea	52 (35.9)	140 (37.3)	35 (53.8)	0.029	0.041
Fever	80 (55.2)	209 (55.7)	40 (61.5)	0.655	0.477
Rhinorrhea, nasal congestion	9 (6.2)	28 (7.5)	2 (3.1)	0.410	0.636
Diarrhoea	25 (17.2)	53 (14.1)	13 (20.0)	0.393	0.931
Vital signs and parameters for oxygen status					
Systolic blood pressure, mmHg	133.3 (±17.1)	137.4 (±20.5)	139.7 (±22.8)	0.048	0.016
Diastolic blood pressure, mmHg	82.3 (±13.7)	83.6 (±13.3)	83.6 (±18.5)	0.607	0.410
Heart rate, /min	88.0 (±17.1)	89.7 (±15.4)	97.1 (±20.6)	0.001	0.001
Respiratory rate, /min	20.2 (±3.5)	20.5 (±3.6)	21.2 (±3.9)	0.168	0.078
SpO_2_, %	94.9 (±4.8)	94.4 (±4.7)	91.5 (±9.2)	<0.001	<0.001
Oxygen requirement, L/min	2.4 (±2.0)	2.6 (±2.0)	4.1 (±3.6)	<0.001	<0.001
Fraction of inspired O_2_ (FiO_2_)	0.30 (±0.08)	0.32 (±0.08)	0.37 (±0.14)	<0.001	<0.001
SpO_2_/FiO_2_ ratio (SF ratio)	324.4 (±50.2)	312.0 (±54.0)	273.0 (±80.7)	<0.001	<0.001
ROX index	16.5 (±3.5)	15.8 (±4.1)	13.4 (±4.7)	<0.001	<0.001
Laboratory indices					
WBC, ×10^3^/µL	3.25 (±0.51)	6.04 (±1.53)	12.96 (±3.62)	<0.001	<0.001
Neutrophil, %	66.3 (±12.7)	72.0 (±12.0)	85.6 (±9.3)	<0.001	<0.001
<1500, 10^3^/µL	16 (11.0)	0	0	<0.001	<0.001
≥7500, 10^3^/µL	0	18 (4.8)	62 (95.4)	<0.001	<0.001
Lymphocyte, %	25.0 (±10.5)	20.1 (±9.4)	9.0 (±6.7)	<0.001	<0.001
<1000, 10^3^/µL	106 (73.1)	160 (42.7)	40 (61.5)	<0.001	<0.001
Neutrophil-to-lymphocyte ratio (NLR)	3.6 (±2.7)	5.0 (±3.8)	16.0 (±17.0)	<0.001	<0.001
Monocyte, %	7.8 (±4.3)	6.8 (±4.7)	4.4 (±3.2)	<0.001	<0.001
Haemoglobin, g/dL	12.7 (±1.8)	13.0 (±1.8)	13.0 (±1.8)	0.201	0.124
Platelet, ×10^3^/µL	143.1 (±53.1)	183.7 (±76.6)	211.4 (±74.7)	<0.001	<0.001
C-reactive protein (CRP), mg/dL	4.6 (±4.6)	7.1 (±6.0)	12.9 (±9.6)	<0.001	<0.001
C-reactive protei*n* > 2, *n* (%)	94 (64.8)	278 (74.1)	56 (86.2)	0.004	0.001
Procalcitonin, ng/mL	0.20 (±0.36)	0.18 (±0.25)	0.61 (±1.85)	<0.001	0.002
Procalcitoni*n* > 0.5, *n* (%)	6 (4.1)	10 (2.7)	10 (15.4)	<0.001	0.009
Lactate dehydrogenase (LDH), U/L	297.2 (±106.4)	329.7 (±141.0)	383.4 (±139.2)	<0.001	<0.001
D-dimer, mg/L	0.95 (±1.10)	1.35 (±2.48)	2.52 (±4.06)	<0.001	<0.001
D-dime*r* > 0.5, *n* (%)	89 (61.4)	256 (68.3)	58 (89.2)	<0.001	<0.001
Troponin I, pg/mL	26.4 (±137.9)	16.9 (±28.7)	26.9 (±37.7)	0.317	0.665
NT-proBNP, pg/mL	528.0 (±63.5)	537.9 (±218.2)	514.4 (±86.8)	0.583	0.844
Blood urea nitrogen (BUN), mg/dL	18.4 (±15.4)	18.7 (±15.1)	26.6 (±17.7)	<0.001	0.005
Creatinine, mg/dL	1.12 (±2.16)	1.10 (±1.79)	1.02 (±0.62)	0.924	0.729
Aspartate aminotransferase (AST), U/L	40.4 (±21.5)	44.7 (±28.9)	55.1 (±31.9)	0.002	0.001
Alanine transaminase (ALT), U/L	26.7 (±17.9)	31.0 (±21.8)	41.2 (±34.3)	<0.001	<0.001
Total bilirubin, mg/dL	0.6 (±0.2)	0.7 (±0.6)	0.8 (±0.4)	0.058	0.017
Prothrombin time (PT), INR	1.05 (±0.06)	1.07 (±0.06)	1.09 (±0.08)	0.001	<0.001
Pneumonia in chest X-ray, *n* (%)	130 (89.7)	347 (92.5)	60 (92.3)	0.556	0.376

Data are presented as *n* (%) or mean ± standard deviation.

*Categorical variables were analysed using Cochran-Armitage test, continuous variables were analysed using linear regression.

### Treatment and clinical outcomes for COVID-19

**Figure 2.**
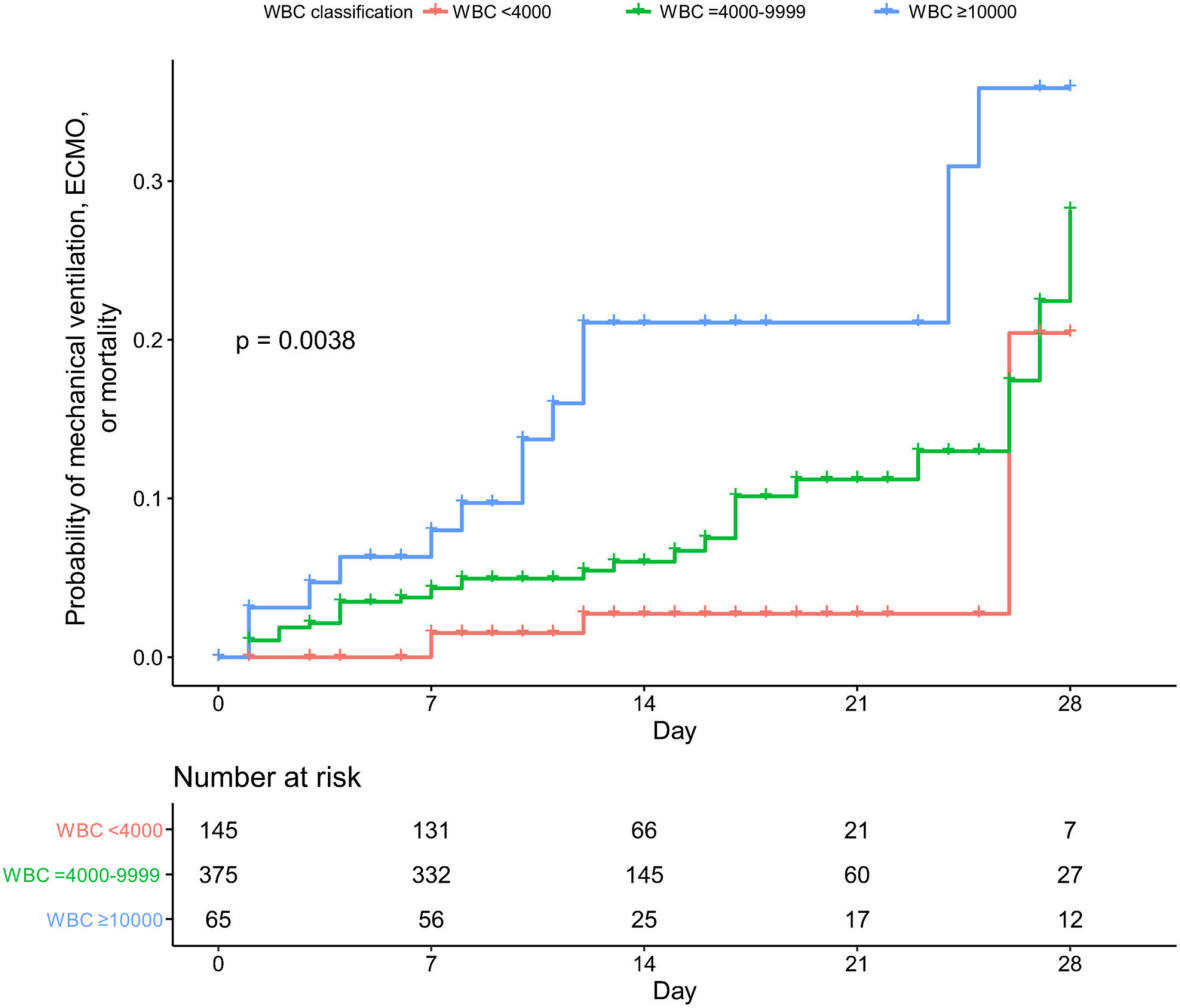
Probability of mechanical ventilation, ECMO, or mortality. Red line (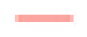) = leukopoenia (WBC count <4000), green line (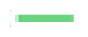) = normal WBC (WBC count = 4000–9999), blue line (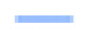) = leukocytosis (WBC count ≥10,000). Log-rank test in comparison with leukopoenia (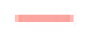) vs. normal WBC (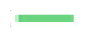) resulted *p*-value of 0.060. Log-rank test in comparison with leukopoenia (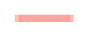) vs. leukocytosis (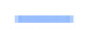) resulted *p*-value of <0.001. Log-rank test in comparison with normal WBC (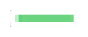) vs. leukocytosis (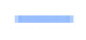) resulted *p*-value of 0.024.

Treatment of COVID-19 infection across the groups showed difference in the duration of steroid therapy; it was the longest in leukocytosis group with the mean of 12.5 days compared to 9.5 days in both leukopoenia and normal WBC group (Table 3). The use of remdesivir or antibiotics was similar across the groups. The hospital length of stay was the longest in leukocytosis group with 31.3 days compared to 16.7 days and 15.7 days in leukopoenia and normal WBC group, respectively. In Kaplan-Meier curve, the composite poor outcome of mechanical ventilation, ECMO and mortality was significantly reduced in leukopoenia group compared to leukocytosis group (log-rank *p*-value < 0.001), but not compared to normal WBC group (log-rank *p*-value = 0.060) (Figure 2).

**Table 3.** Treatment and clinical outcomes according to different white blood cell counts.

	WB*C* < 4000 (*n* = 145)	WBC 4000–9999 (*n* = 375)	WB*C* ≥ 10,000 (*n* = 65)	*p*-Value	*p*-Trend*
Treatment					
Lopinavir/Ritonavir, *n* (%)	2 (1.4)	4 (1.1)	0 (0.0)	0.651	0.408
Hydroxychloroquine, *n* (%)	1 (0.7)	3 (0.8)	0 (0.0)	0.770	0.697
Remdesivir, *n* (%)	126 (86.9)	320 (85.3)	57 (87.7)	0.823	0.965
Antibiotics, *n* (%)	114 (78.6)	300 (80.0)	50 (76.9)	0.828	0.924
Duration of steroids, days	9.5 (±6.3)	9.5 (±6.1)	12.5 (±9.2)	0.002	0.020
Cumulative dose of steroids^a^, mg	386.8 (±337.4)	420.8 (±356.5)	493.8 (±387.7)	0.136	0.057
High flow nasal cannula, *n* (%)	37 (25.5)	133 (35.5)	40 (61.5)	<0.001	<0.001
Composite poor outcome, *n* (%)	7 (4.8)	40 (10.7)	17 (26.2)	<0.001	<0.001
Mechanical ventilation	1 (0.7)	21 (5.6)	11 (16.9)	<0.001	<0.001
ECMO	0 (0.0)	5 (1.3)	4 (6.2)	0.003	0.003
Mortality	6 (4.1)	25 (6.7)	8 (12.3)	0.090	0.037
Hospital length of stay, days	16.7 (±30.8)	15.7 (±12.3)	31.3 (±65.7)	<0.001	0.012

^a^Dose is based on an equivalent dose of methylprednisolone.

Data are presented as *n* (%) or mean ± standard deviation.

*Categorical variables were analysed using Cochran-Armitage test, continuous variables were analysed using linear regression.

ECMO: extracorporeal membrane oxygenation.

### Multivariable analysis to evaluate risk factors of composite poor outcome

In the univariable Cox regression analysis, leukopoenia group showed a lower hazard ratio (HR) for composite poor outcome compared to normal WBC group. In addition, age greater than 65 years, connective tissue disease, febrile manifestation, a higher systolic blood pressure, procalcitonin greater than 0.5 ng/mL, a lower ROX index, neutrophilia (≥7500, 103/µL), NLR, a lower haemoglobin, and a lower platelet count were associated with a higher HR for composite poor outcome. In the multivariable Cox regression analysis, leukopoenia group was significantly associated with a lower risk of composite poor outcome compared to normal WBC group (adjusted HR (aHR)=0.32, 95% CI = 0.14–0.76, *p*-value = 0.009) and leukocytosis group (aHR = 0.30, 95% CI = 0.12–0.78, *p*-value = 0.013) in COVID-19 patients who were treated with systemic corticosteroids (Table 4). On the other hand, leukocytosis group was not associated with composite poor outcome (aHR 1.08, 95% CI 0.52–2.21, *p*-value = 0.845) compared to normal WBC group. In the multivariable analyses according to the differential count of white blood cells, neutrophilia was related with a higher risk of composite poor outcome, but there was no association between neutropenia or lymphopenia and composite poor outcome (supplemental Table S3 and S4). In addition, we found no association between NLR and composite poor outcome in the multivariable analysis (supplemental Table S5). Nomogram of variables on composite poor outcome is described in supplemental Figure S2.

**Table 4.** Cox regression analysis to identify risk factors of mechanical ventilation, ECMO, or mortality.

	uHR (95% CI)	*p*-Value	aHR (95% CI)	*p*-Value
Ag*e* > 65 years	2.67 (1.40–5.09)	0.003	2.15 (1.09–4.25)	0.028
Comorbidity				
COPD	2.13 (0.98–4.61)	0.055	1.31 (0.50–3.46)	0.577
Cardiovascular disease	1.76 (0.97–3.21)	0.064	1.57 (0.82–3.00)	0.171
Connective tissue disease	3.62 (1.29–10.1)	0.014	4.79 (1.47–15.62)	0.009
Clinical characteristics				
Fever	3.46 (1.91–6.30)	<0.001	3.14 (1.63–6.03)	<0.001
Systolic blood pressure, mmHg	1.02 (1.003–1.03)	0.011	1.01 (0.999–1.02)	0.073
ROX index	0.95 (0.90–0.99)	0.028	0.93 (0.88–0.99)	0.019
Laboratory indices				
WBC (ref: 4000–9999, 10^3^/µL)	–	–		
<4000, 10^3^/µL	0.41 (0.18–0.89)	0.024	0.34 (0.15–0.80)	0.014
≥10,000, 10^3^/µL	1.61 (0.89–2.91)	0.117	0.46 (0.14–1.50)	0.195
Neutrophi*l* ≥ 7500, 10^3^/µL	2.43 (1.43–4.12)	0.001	2.66 (0.83–8.51)	0.099
NLR	1.02 (1.01–1.04)	0.011	1.00 (0.97–1.04)	0.885
Haemoglobin, g/dL	0.85 (0.75–0.96)	0.010	0.81 (0.71–0.92)	<0.001
Platelet count, 10^3^/µL	0.99 (0.99–0.99)	0.013	0.99 (0.989–0.998)	0.005
Procalcitoni*n* > 0.5 ng/mL	3.64 (1.86–7.11)	<0.001	2.57 (1.16–5.71)	0.021

aHR: adjusted hazard ratio; CI: confidence interval; COPD: chronic obstructive pulmonary disease; NLR: neutrophil-lymphocyte ratio; uHR: unadjusted hazard ratio.

## Discussion

Our study investigated treatment response of systemic corticosteroid therapy in COVID-19 patients who required oxygen therapy according to the blood leukocyte counts. Leukopoenia group showed a significantly lower hazard of composite poor outcome consisting of mechanical ventilation, ECMO and mortality compared to normal WBC or leukocytosis group in multivariable analysis. Leukocytosis group showed an increased risk of composite poor outcome in survival analysis, but did not increase the hazard of composite poor outcome compared to normal WBC group after adjustment for disease severity. Neutrophilia was related with an increased hazard of composite poor outcome, while neutropenia, lymphopenia, and NLR were not associated with composite poor outcome in multivariable analyses. Therefore, a better treatment response may be expected in the patients with leukopoenia who were treated with systemic corticosteroids for severe COVID-19 pneumonia.

There has been a growing interest in identifying clinical sub-phenotypes of COVID-19 pneumonia or COVID-19-related ARDS for establishing individualised therapy. Especially, phenotyping with various inflammatory profiles has been expected useful in predicting treatment response to systemic corticosteroid in COVID-19. A previous study conducted cluster analysis using inflammatory biomarkers to investigate hyper- and hypo-inflammatory phenotypes in critically ill COVID-19 patients who used systemic corticosteroids [5]. This study reported that a better efficacy of systemic corticosteroid therapy was observed in the patient group with hyper-inflammatory phenotypes. Although systemic corticosteroid treatment was not randomised, another study conducted latent class analysis to find a better response to systemic corticosteroid in the patients with hyper-inflammatory phenotypes [8]. COVID-19 can cause systemic hyper-inflammation with features of cytokine storm syndrome [12,13], and systemic corticosteroids are thought to be effective because they down-regulate systemic hyper-inflammatory response.

Our study figured out that there may be a difference in the response to systemic corticosteroids according to the WBC profiles, which is one of the important inflammatory markers. WBC count reflected the immunologic response in COVID-19 that was different from other respiratory viral infections and was related to the prognosis in COVID-19 patients. The leukocyte expression profiles were different between hypo- and hyper-inflammatory ARDS [14]. In our study, the hazard of composite poor outcome was lower in leukopoenia group compared to normal WBC or leukocytosis group in severe COVID-19 patients who used systemic corticosteroid. Leukopoenia has been frequently found in the hyperinflammatory syndromes including secondary haemophagocytic lymphohistiocytosis, macrophage activation syndrome, and cytokine release syndrome [15]. Therefore, leukopoenia may be a biologic marker for hyper-inflammatory phenotype in COVID-19 with a better response to systemic corticosteroid [16–18].

Lymphopenia has been suggested as a poor prognostic factor in COVID-19. Compared to the cytokine storm syndromes caused by other diseases, COVID-19 induced hyper-inflammation is different in its haematologic manifestation as decreased lymphocyte count was related with poor outcomes [19,20]. The mechanism behind the development of lymphopenia in COVID-19 is suggested to be multi-factorial: direct cell infection by the virus, indirect destruction by hyper-inflammation caused *via* IL-6 and TNF-α pathways, and lymphocytes recruitment to the inflammatory lung tissues [21]. Our study showed that lymphopenia was not a prognostic factor in COVID-19 patients who used systemic corticosteroids. Systemic corticosteroids are reported to increase lymphocyte counts in clinically responsive COVID-19 patients [22]. Immunomodulating effect of systemic corticosteroids may recover T-cell dysregulation and or alleviate the inflammatory process [23,24].

Neutropenia was only found in leukopoenia group but neutrophilia was not. Low neutrophil count was related with a higher mortality, and there was an insignificant trend of decreasing mortality after administration of systemic corticosteroid in COVID-19 patients with neutropenia [25]. In our study, neutropenia was not significantly associated with the hazard of composite poor outcome after treatment with systemic corticosteroid. Neutrophilia is another haematologic marker associated with poor outcome in COVID-19 [26–28]. Neutrophilia is related with the increased release of proinflammatory cytokines, activation of neutrophil, induction of oxidative stress, and formation of neutrophil extracellular traps, resulting in inflammatory lung injury [29]. Neutrophilia has been a predictive marker of progression of ARDS associated with a systemic hyper-inflammatory response [29]. However, even though systemic corticosteroid was administered, the patients with neutrophilia showed a higher hazard of the composite poor outcome in our study. Therefore, additional therapeutic intervention combined with systemic corticosteroid may be necessary to further modify dysregulation of excessive inflammatory response to COVID-19 pneumonia in the patients with neutrophilia.

The present study has several limitations. First, our study is a retrospective cohort study in a single medical centre. For generalisation, external validation within a larger cohort is needed. Second, different prognosis according to WBC counts may be related with a different severity of COVID-19, rather than the response to systemic corticosteroid. Therefore, we included only severe COVID-19 patients who required oxygen therapy and analysed them with adjustment for age, comorbidities, ROX index, and other laboratory findings. Even considering this limitation, lymphopenia is speculated as a biologic indicator for favourable outcomes in COVID-19 patients treated with systemic corticosteroid. Third, to properly estimate the different efficacy of systemic corticosteroid according to WBC counts, clinical outcomes should be compared according to whether or not steroids were administered in each group. However, it is not feasible in real-world to compare prognosis between the patients who used steroids and those who did not because of the considerable difference in disease severity between them. In addition, random allocation for steroid administration according to specific biomarkers in the patients with severe COVID-19 who requires oxygen is considered ethically violated under current treatment consensus. Therefore, our study evaluated clinical outcomes as an indirect indicator for treatment response to systemic corticosteroids in severe COVID-19 patients. Fourth, further *in vivo* or *in vitro* study is needed to elucidate the pathophysiological background behind WBC changes with corticosteroid therapy during severe COVID-19 infection.

## Conclusion

Leukopoenia group showed a lower hazard for composite poor outcome compared to normal WBC or leukocytosis group in severe COVID-19 patients who were treated with systemic corticosteroids. Leukopoenia may be a potential biomarker for better response to systemic corticosteroid therapy in COVID-19 pneumonia.

## Supplementary Material

Supplemental Material

Supplemental Material

Supplemental Material

## Data Availability

The datasets used and/or analysed during the current study are available from the corresponding author on reasonable request.
