# Supplementary figures and images for: Different treatment response to systemic corticosteroids according to white blood cell counts in severe COVID-19 patients
Source: Ann Med. 2022 Dec 1;54(1):2998–3006. doi: 10.1080/07853890.2022.2137736 (PMC9721443; doi:10.1080/07853890.2022.2137736)

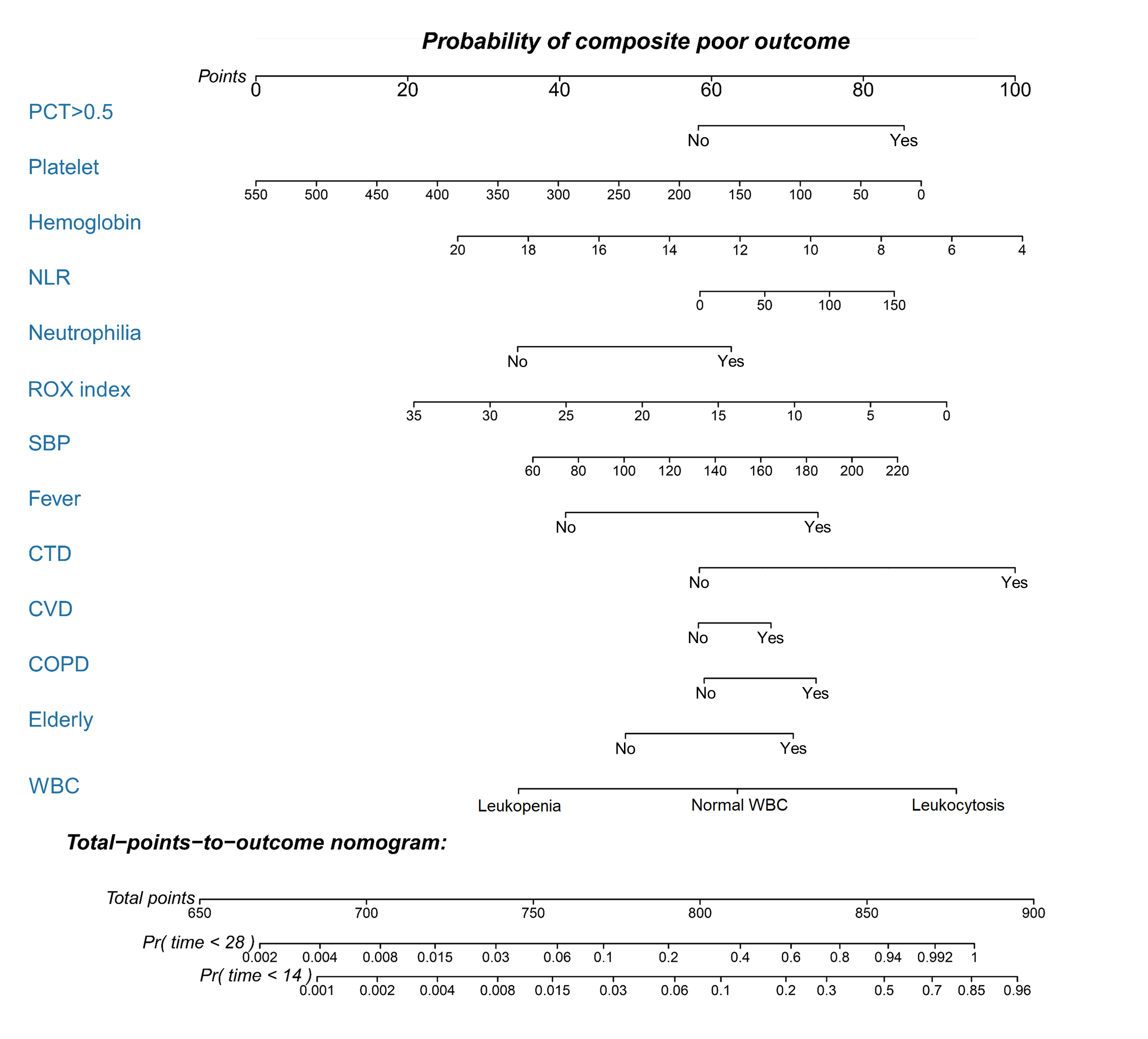

Supplement: Supplemental Material [file IANN_A_2137736_SM3755.tif]

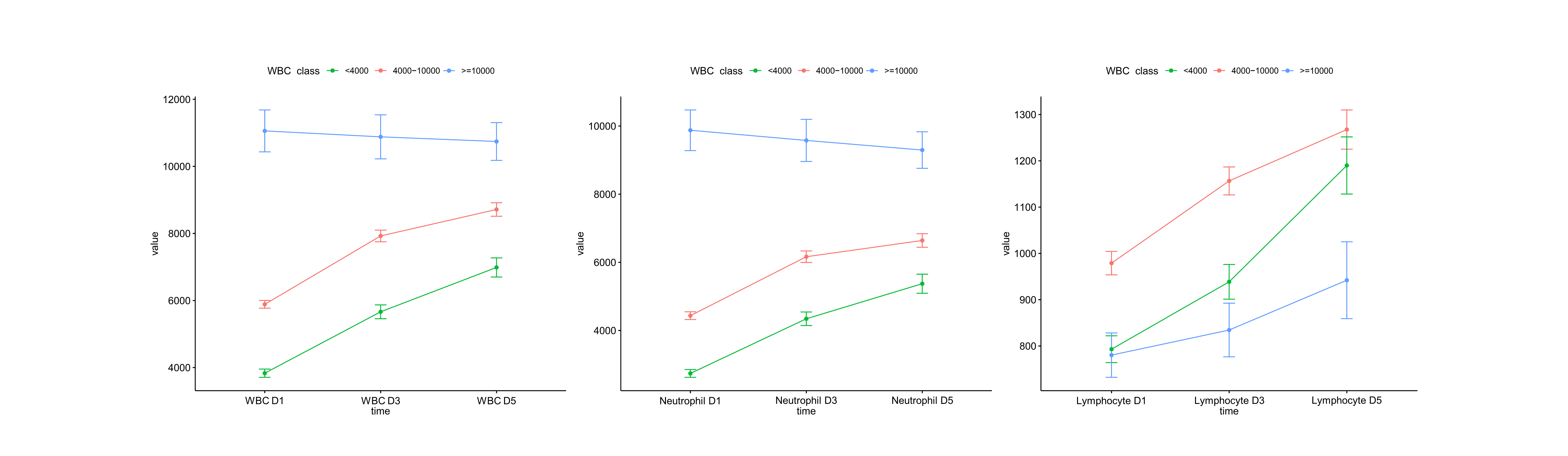

Supplement: Supplemental Material [file IANN_A_2137736_SM3754.tif]
