# Supplementary material for: Different treatment response to systemic corticosteroids according to white blood cell counts in severe COVID-19 patients
Source: Ann Med. 2022 Dec 1;54(1):2998–3006. doi: 10.1080/07853890.2022.2137736 (PMC9721443; doi:10.1080/07853890.2022.2137736)
Supplement: Supplemental Material [file IANN_A_2137736_SM3749.docx]

**Supplementary information legends**

**Table S1.** Baseline demographic characteristics according to composite poor outcome

|  | **No composite poor outcome (n = 521)** | **Positive composite poor outcome (n = 64)** | **uHR (95% CI)** | ***P*-value** |
| --- | --- | --- | --- | --- |
| **Age, years** | 68.0 (±14.1) | 77.2 (±10.7) | 1.03 (1.01-1.05) | 0.014 |
| **Age > 65, n (%)** | 234 (44.9) | 52 (81.2) | 2.67 (1.40-5.09) | 0.003 |
| **Female, n (%)** | 235 (45.1) | 27 (42.2) | 0.76 (0.46-1.25) | 0.276 |
| **Body mass index, kg/m^2^** | 24.7 (±4.1) | 23.7 (3±3.7) | 0.95 (0.90-1.01) | 0.089 |
| **Ever smoker, n (%)** | 37 (7.1) | 11 (17.2) | 1.66 (0.85-3.22) | 0.135 |
| **Smoking intensity, pack-years** | 1.6 (±7.3) | 5.3 (±15.9) | 1.01 (1.00-1.03) | 0.167 |
| **Comorbidity, n (%)** |  |  |  |  |
| Hypertension | 263 (50.5) | 36 (56.2) | 0.96 (0.58-1.60) | 0.887 |
| Diabetes mellitus | 153 (29.4) | 26 (40.6) | 1.18 (0.71-1.96) | 0.532 |
| COPD | 9 (1.7) | 8 (12.5) | 2.13 (0.98-4.61) | 0.055 |
| Asthma | 18 (3.5) | 2 (3.1) | 0.91 (0.22-3.73) | 0.895 |
| ILD | 1 (0.2) | 0 (0.0) | - |  |
| Tuberculosis | 16 (3.1) | 5 (7.8) | 1.91 (0.76-4.79) | 0.167 |
| Lung cancer | 5 (1.0) | 2 (3.1) | 0.76 (0.18-3.24) | 0.713 |
| Other malignancy | 44 (8.4) | 8 (12.5) | 1.91 (0.90-4.06) | 0.092 |
| Chronic kidney disease | 27 (5.2) | 5 (7.8) | 1.17 (0.47-2.94) | 0.736 |
| Chronic liver disease | 18 (3.5) | 1 (1.6) | 0.59 (0.08-4.29) | 0.604 |
| Cardiovascular disease | 59 (11.3) | 14 (21.9) | 1.76 (0.97-3.21) | 0.064 |
| Cerebrovascular disease | 67 (12.9) | 9 (14.1) | 0.68 (0.33-1.40) | 0.092 |
| Cognitive disorder | 6 (1.2) | 0 (0.0) | - |  |
| Connective tissue disease | 11 (2.1) | 4 (6.2) | 3.62 (1.29-10.1) | 0.014 |

Data are presented as n (%) or mean ± standard deviation.

uHR, unadjusted hazard ratio

**Table S2.** Clinical manifestation according to composite poor outcome

|  | **No composite poor outcome (n = 521)** | **Positive composite poor outcome (n = 64)** | **uHR (95% CI)** | ***P*-value** |
| --- | --- | --- | --- | --- |
| **Symptomatic manifestation, n (%)** |  |  |  |  |
| Smell or taste abnormality | 97 (18.6) | 9 (14.1) | 1.01 (0.49-2.05) | 0.990 |
| Myalgia | 166 (31.9) | 10 (15.6) | 0.49 (0.25-0.96) | 0.038 |
| Sore throat | 108 (20.7) | 9 (14.1) | 1.01 (0.49-2.07) | 0.974 |
| Cough | 330 (63.3) | 41 (64.1) | 1.51 (0.90-2.54) | 0.117 |
| Sputum | 233 (44.7) | 33 (51.6) | 1.33 (0.81-2.18) | 0.257 |
| Chest discomfort | 63 (12.1) | 12 (18.8) | 1.89 (1.004-3.55) | 0.049 |
| Dyspnea | 186 (35.7) | 41 (64.1) | 2.84 (1.70-4.77) | <0.001 |
| Fever | 279 (53.6) | 50 (78.1) | 3.46 (1.91-6.30) | <0.001 |
| Rhinorrhea, nasal congestion | 34 (6.5) | 5 (7.8) | 1.54 (0.61-3.87) | 0.356 |
| Diarrhea | 67 (12.9) | 24 (37.5) | 2.90 (1.73-4.87) | <0.001 |
| **Vital signs and parameters for oxygen status** |  |  |  |  |
| Systolic blood pressure, mmHg | 135.4 (±18.9) | 146.3 (±26.0) | 1.02 (1.003-1.03) | 0.011 |
| Diastolic blood pressure, mmHg | 83.1 (±12.9) | 84.8 (±21.5) | 1.00 (0.99-1.02) | 0.641 |
| Heart rate, /min | 90.0 (±15.8) | 90.9 (±22.8) | 1.00 (0.99-1.01) | 0.748 |
| Respiratory rate, /min | 20.4 (±3.4) | 21.6 (±4.7) | 1.06 (1.00-1.12) | 0.071 |
| SpO_2_, % | 94.5 (±5.0) | 91.7 (±7.9) | 0.99 (0.96-1.02) | 0.455 |
| Oxygen requirement, L/min | 2.6 (±2.1) | 4.0 (±3.3) | 1.04 (0.97-1.10) | 0.282 |
| Fraction of inspired O_2_ (FiO_2_) | 0.31 (±0.08) | 0.37 (±0.13) | 2.40 (0.49-11.9) | 0.282 |
| SpO_2_/FiO_2_ ratio (SF ratio) | 315.5 (±54.1) | 271.9 (±75.5) | 0.99 (0.99-1.00) | 0.033 |
| ROX index | 16.0 (±3.9) | 13.4 (±4.9) | 0.95 (0.90-0.99) | 0.028 |
| **Laboratory indices** |  |  |  |  |
| WBC, x10^3^/uL | 5.87 (±2.99) | 8.16 (±4.07) | 1.0001 (1.00-1.0001) | 0.012 |
| WBC <4,000 uL, n (%) | 138 (26.5) | 7 (10.9) | 0.41 (0.18-0.89) | 0.024 |
| WBC ≥10,000 uL, n (%) | 48 (9.2) | 17 (26.6) | 1.61 (0.89-2.91) | 0.117 |
| Neutrophil, % | 71.5 (±13.0) | 77.0 (±12.1) | 1.03 (1.01-1.05) | 0.005 |
| <1,500, 10^3^/uL | 15 (2.9) | 1 (1.6) | 0.57 (0.08-4.16) | 0.584 |
| ≥7,500, 10^3^/uL | 59 (11.3) | 21 (32.8) | 2.43 (1.43-4.12) | 0.001 |
| Lymphocyte, % | 20.6 (±10.4) | 15.7 (±9.6) | 0.96 (0.94-0.99) | 0.002 |
| <1,000, 10^3^/uL | 272 (52.2) | 34 (53.1) | 1.03 (0.63-1.68) | 0.921 |
| Neutrophil-to-lymphocyte ratio (NLR) | 5.4 (±6.9) | 9.1 (±10.5) | 1.02 (1.01-1.04) | 0.011 |
| Monocyte, % | 6.8 (±4.7) | 6.1 (±3.9) | 0.94 (0.88-1.01) | 0.094 |
| Hemoglobin, g/dL | 13.0 (±1.8) | 12.3 (±1.6) | 0.85 (0.75-0.96) | 0.010 |
| Platelet, x10^3^/uL | 179.9 (±75.8) | 150.8 (±54.1) | 0.99 (0.99-0.999) | 0.013 |
| C-reactive protein (CRP), mg/dL | 7.0 (±6.4) | 8.8 (±7.7) | 1.02 (0.99-1.05) | 0.187 |
| C-reactive protein > 2, n (%) | 373 (71.6) | 55 (85.9) | 0.43 (0.21-0.87) | 0.019 |
| Procalcitonin, ng/mL | 0.19 (±0.26) | 0.59 (±1.90) | 1.07 (0.96-1.19) | 0.258 |
| Procalcitonin > 0.5, n (%) | 15 (2.9) | 11 (17.2) | 3.64 (1.86-7.11) | <0.001 |
| Lactate dehydrogenase (LDH), U/L | 322.6 (±133.7) | 368.8 (±139.7) | 1.00 (1.00-1.00) | 0.091 |
| D-dimer, mg/L | 1.27 (±2.18) | 2.28 (±4.21) | 1.07 (1.02-1.14) | 0.014 |
| D-dimer > 0.5, n (%) | 351 (67.4) | 52 (81.2) | 0.77 (0.41-1.47) | 0.434 |
| Troponin I, pg/mL | 19.4 (±76.3) | 28.7 (±42.0) | 1.00 (1.00-1.00) | 0.214 |
| NT-proBNP, pg/mL | 530.7 (±171.1) | 549.6 (±241.3) | 1.00 (1.00-1.00) | 0.590 |
| Blood urea nitrogen (BUN), mg/dL | 18.6 (±14.7) | 27.0 (±20.9) | 1.01 (1.001-1.02) | 0.026 |
| Creatinine, mg/dL | 1.1 (±1.8) | 1.5 (±1.9) | 1.10 (1.01-1.19) | 0.022 |
| Aspartate aminotransferase (AST), U/L | 44.6 (±28.1) | 46.5 (±25.9) | 1.00 (0.99-1.01) | 0.639 |
| Alanine transaminase (ALT), U/L | 31.4 (±23.2) | 28.9 (±21.3) | 0.99 (0.98-1.01) | 0.385 |
| Total bilirubin, mg/dL | 0.7 (±0.5) | 0.7 (±0.3) | 1.03 (0.59-1.82) | 0.915 |
| Prothrombin time (PT), INR | 1.07 (±0.06) | 1.08 (±0.07) | 1.61 (0.07-35.8) | 0.765 |
| **Pneumonia in chest X-ray, n (%)** | 480 (92.1) | 57 (89.1) | 0.81 (0.37-1.80) | 0.606 |

Data are presented as n (%) or mean ± standard deviation.

uHR, unadjusted hazard ratio

**Table S3.** Association between neutrophil counts and poor composite outcome

|  | **uHR (95% CI)** | ***P*-value** | **aHR (95% CI)** | ***P*-value** |
| --- | --- | --- | --- | --- |
| **Age > 65 years** | 2.67 (1.40-5.09) | 0.003 | 2.33 (1.19-4.52) | 0.013 |
| **Comorbidity** |  |  |  |  |
| COPD | 2.13 (0.98-4.61) | 0.055 | 1.39 (0.57-3.41) | 0.466 |
| Cardiovascular disease | 1.76 (0.97-3.21) | 0.064 | 1.44 (0.76-2.74) | 0.265 |
| Connective tissue disease | 3.62 (1.29-10.1) | 0.014 | 3.52 (1.15-10.77) | 0.028 |
| **Clinical characteristics** |  |  |  |  |
| Fever | 3.46 (1.91-6.30) | <0.001 | 3.67 (1.90-7.09) | <0.001 |
| Systolic blood pressure, mmHg | 1.02 (1.003-1.03) | 0.011 | 1.01 (1.001-1.02) | 0.030 |
| ROX index | 0.95 (0.90-0.99) | 0.028 | 0.93 (0.88-0.99) | 0.013 |
| **Laboratory indices** |  |  |  |  |
| Neutrophil (ref: ≥1,500 & <7,500, 10^3^/uL) | - | - | - | - |
| <1,500, 10^3^/uL | 0.71 (0.10-5.17) | 0.734 | 1.49 (0.20-11.21) | 0.697 |
| ≥7,500, 10^3^/uL | 2.40 (1.41-4.09) | 0.001 | 2.05 (1.05-3.99) | 0.034 |
| Hemoglobin, g/dL | 0.85 (0.75-0.96) | 0.010 | 0.82 (0.72-0.93) | 0.001 |
| Platelet count, 10^3^/uL | 0.99 (0.99-0.99) | 0.013 | 0.99 (0.99-1.00) | 0.013 |
| Procalcitonin > 0.5 ng/mL | 3.64 (1.86-7.11) | <0.001 | 2.31 (1.12-4.76) | 0.024 |

aHR, adjusted hazard ratio; CI, confidence interval; COPD, chronic obstructive pulmonary disease; uHR, unadjusted hazard ratio

**Table S4.** Association between lymphocyte counts and poor composite outcome

|  | **uHR (95% CI)** | ***P*-value** | **aHR (95% CI)** | ***P*-value** |
| --- | --- | --- | --- | --- |
| **Age > 65 years** | 2.67 (1.40-5.09) | 0.003 | 2.08 (1.07-4.06) | 0.031 |
| **Comorbidity** |  |  |  |  |
| COPD | 2.13 (0.98-4.61) | 0.055 | 1.45 (0.61-3.46) | 0.397 |
| Cardiovascular disease | 1.76 (0.97-3.21) | 0.064 | 1.49 (0.78-2.85) | 0.227 |
| Connective tissue disease | 3.62 (1.29-10.1) | 0.014 | 4.18 (1.42-12.28) | 0.009 |
| **Clinical characteristics** |  |  |  |  |
| Fever | 3.46 (1.91-6.30) | <0.001 | 4.18 (2.18-8.00) | <0.001 |
| Systolic blood pressure, mmHg | 1.02 (1.003-1.03) | 0.011 | 1.01 (1.001-1.02) | 0.049 |
| ROX index | 0.95 (0.90-0.99) | 0.028 | 0.91 (0.86-0.96) | 0.001 |
| **Laboratory indices** |  |  |  |  |
| Lymphocyte (ref: ≥1,000, 10^3^/uL) | - | - | - | - |
| <1,000, 10^3^/uL | 0.98 (0.60-1.60) | 0.921 | 0.64 (0.35-1.16) | 0.139 |
| Hemoglobin, g/dL | 0.85 (0.75-0.96) | 0.010 | 0.83 (0.73-0.94) | 0.003 |
| Platelet count, 10^3^/uL | 0.99 (0.99-0.99) | 0.013 | 1.00 (0.99-1.00) | 0.022 |
| Procalcitonin > 0.5 ng/mL | 3.64 (1.86-7.11) | <0.001 | 3.16 (1.56-6.41) | 0.001 |

aHR, adjusted hazard ratio; CI, confidence interval; COPD, chronic obstructive pulmonary disease; uHR, unadjusted hazard ratio

**Table S5.** Association between neutrophil-to-lymphocyte ratio and poor composite outcome

|  | **uHR (95% CI)** | ***P*-value** | **aHR (95% CI)** | ***P*-value** |
| --- | --- | --- | --- | --- |
| **Age > 65 years** | 2.67 (1.40-5.09) | 0.003 | 2.35 (1.17-4.70) | 0.016 |
| **Comorbidity** |  |  |  |  |
| COPD | 2.13 (0.98-4.61) | 0.055 | 1.79 (0.76-4.22) | 0.184 |
| Cardiovascular disease | 1.76 (0.97-3.21) | 0.064 | 1.45 (0.76-2.78) | 0.260 |
| Connective tissue disease | 3.62 (1.29-10.1) | 0.014 | 5.14 (1.76-15.01) | 0.003 |
| **Clinical characteristics** |  |  |  |  |
| Fever | 3.46 (1.91-6.30) | <0.001 | 4.12 (2.16-7.86) | <0.001 |
| Systolic blood pressure, mmHg | 1.02 (1.003-1.03) | 0.011 | 1.01 (1.00-1.02) | 0.031 |
| ROX index | 0.95 (0.90-0.99) | 0.028 | 0.93 (0.88-0.99) | 0.015 |
| **Laboratory indices** |  |  |  |  |
| Neutrophil-to-lymphocyte ratio | 1.02 (1.01-1.04) | 0.011 | 1.02 (0.99-1.04) | 0.162 |
| Hemoglobin, g/dL | 0.85 (0.75-0.96) | 0.010 | 0.84 (0.75-0.94) | 0.004 |
| Platelet count, 10^3^/uL | 0.99 (0.99-0.99) | 0.013 | 1.00 (0.99-1.00) | 0.023 |
| Procalcitonin > 0.5 ng/mL | 3.64 (1.86-7.11) | <0.001 | 2.23 (1.02-4.86) | 0.042 |

aHR, adjusted hazard ratio; CI, confidence interval; COPD, chronic obstructive pulmonary disease; uHR, unadjusted hazard ratio
